# Comment on “Metal–organic green dye: chemical and physical insight into a modified Zn-benzoporphyrin for dye-sensitized solar cells” by G. Zanotti, N. Angelini, G. Mattioli, A. M. Paoletti, G. Pennesi, G. Rossi, D. Caschera, L. de Marcoc and G. Giglide, *RSC Adv.*, 2016, **6**, 5123

**DOI:** 10.1039/c8ra00213d

**Published:** 2018-05-11

**Authors:** Ronald P. Steer

**Affiliations:** Department of Chemistry, University of Saskatchewan Saskatoon SK Canada S7N5C9 ron.steer@usask.ca

## Abstract

The emission spectroscopy and photophysics reported in the title paper are shown to be untenable in light of previously reported experimental results and widely accepted theories of electronic excited state relaxation. The published results strongly suggest that the newly synthesized dye is contaminated with one of more highly fluorescent impurities.

## Abstract 2

The emission spectroscopy and photophysics reported in the title paper are shown to be untenable in light of previously reported experimental results and widely accepted theories of electronic excited state relaxation.

## Introduction

The authors of the title paper^1^ report the synthesis of a zinc metalloporphyrin derivative, 5,10,15-(triphenyl),20-[ethynyl-(4-carboxy)phenyl]tetrabenzoporphyrinate Zn(ii) (PETBP), which they have subjected to spectroscopic, electrochemical and photophysical examination. They claim that the results suggest that this compound should be a useful primary absorber to incorporate into dye-sensitized solar cells (DSSCs). While the UV-visible absorption spectrum of the synthesized material appears to be qualitatively consistent with the PETBP structure, the reported emission spectra and photophysical data indicate beyond doubt that the emission is dominated by impurities.

## Discussion

The authors cite a recent article^2^ entitled “Concerning correct and incorrect assignments of Soret (S_2_–S_0_) fluorescence in porphyrinoids: a short critical review” to support their assignments of the fluorescence observed when the newly synthesized material in solution is excited at 460 nm. This review article outlines the criteria that should be applied when assigning emission excited in the strong, fully electric dipole allowed Soret band(s) characteristic of all porphyrinoids. Soret excitation of pure metalloporphyrins containing d^0^ or d^10^ metal ions of low atomic mass results in the spectroscopic and photophysical properties that are outlined briefly in the following paragraph.

As a result of rapid and near quantitative S_2_–S_1_ internal conversion, the quantum yield of S_1_–S_0_ fluorescence is almost identical for both direct Q band excitation and indirect excitation in the Soret band. However, owing to their large S_2_ radiative rates and large S_2_–S_1_ electronic energy spacings, highly purified metalloporphyrins such as the model compounds zinc tetraphenylporphyrin (ZnTPP), zinc tetrabenzoporphyrin (ZnTBP) and zinc tetraphenyltetrabenzoporphyrin (ZnTPTBP)^3^ do exhibit weak but measurable fluorescence from the initially-excited S_2_ state. The quantum yields of this “anomalous” S_2_–S_0_ fluorescence are of the order of 10^−3^ and the S_2_ excited state lifetimes are of the order of 1 ps.^3^ Consistent with the rigid structures of the macrocycles, these pure compounds in dilute inert solution exhibit only one strong Gaussian S_0_–S_2_ absorption band in the Soret region and a single, S_2_–S_0_ fluorescence band that is a mirror-image of the Soret absorption with a Stokes shift of no more than 10 nm. Both the S_2_ and S_1_ fluorescence decays are monoexponential when the metalloporphyrins are pure, exhibiting lifetimes of the order of 1 ps and a few ns respectively. Substitution of one of the *meso*-phenyl groups by an ethynyl-(4-carboxy)phenyl moiety does lower the substitutional symmetry of the macrocycle but, as with many other such substituted metalloporphyrins,^4,5^ this does not change these spectroscopic and photophysical properties drastically.

In contrast, the reported emission spectrum of PETBP (Fig. 2 in ref. 1) consists of three readily distinguishable features; two of roughly equal intensity with maxima at *ca.* 500 nm and 530 nm, together with a substantially weaker band with a maximum at 663 nm and an accompanying barely discernible feature at *ca.* 725 nm. The spacing between the two latter features, *ca.* 1300 cm^−1^ (not reported), and the quoted Stokes shift of 8 nm are consistent with metalloporphyrin Q band emission, as assigned by the authors. However, the mid-visible emission is clearly not a mirror image of the absorption in the Soret region. Moreover, the observed Stokes shift of the mid-visible emission maximum at *ca.* 500 nm, relative to the corresponding Soret absorption maximum, 456 nm, is much too large for a rigid macrocycle such as PETBP.

The integrated emission intensity of the two strong bands in the mid-visible, assigned by the authors to S_2_–S_0_ fluorescence, is more than twenty times larger than the integrated intensity of the weak Q fluorescence band in the red. The integrated absorption intensity of PETBP's Soret band will be about the same as that of the model ZnTBP and ZnTPTBP compounds,^3,5^ so the radiative rates of the S_2_ states will be about the same for all three of these zinc metalloporphyrins. Therefore, the much larger emission intensity observed by the authors and assigned to S_2_–S_0_ fluorescence of PETBP, if true, must result in an assigned S_2_–S_0_ fluorescence quantum yield > 10^−2^. This will result in an S_2_ excited state lifetime greater than several tens of picoseconds. However, the S_2_–S_1_ electronic energy gap of PETBP, *ca.* 6600 cm^−1^ (from the absorption spectra, but not reported), is about the same as those of ZnTBP and ZnTPTBP, whose S_2_ lifetimes in solution are *ca.* 1 ps.^3^ This electronic energy gap controls the rate of S_2_ decay *via* the magnitudes of the Franck–Condon factors for the S_2_–S_1_ radiationless transition. These Franck–Condon factors fall off approximately exponentially with increasing spacing between the two coupled electronic states (the energy gap law^3^). If the authors' assignment of the emissions with maxima at 500 nm and 530 nm is correct, PETBP would be wildly different from all other anomalously fluorescent porphyrinoids.

A simple, but better explanation for the authors' observation of a rather intense emission in the mid-visible region is that the sample of PETBP employed is contaminated with a small mole fraction of highly fluorescent impurities that absorb in the porphyrin Soret region. The authors detailed S_1_ fluorescence decay data provided in the ESI provides credible support for this suggestion. In their article, the authors provide only a single constant for the rate of S_1_ decay in solution; 4.6 ns. However, the ESI reports three decay constants of 6.5 ns, 1.4 ns, and 0.23 ns with assigned populations of 60%, 29% and 11% respectively (these data provide a weighted average of 4.3 ns). There is no reason why a tri-exponential function should be required to fit the S_1_ fluorescence decay of a pure zinc metalloporphyrin such as PETBP in dilute solution at room temperature. Rather the tri-exponential fit is highly indicative of impurities whose S_1_–S_0_ fluorescence spectra tail into the porphyrin Q band emission region as shown in Fig. 2.

A second data set in the ESI also supports this explanation of impurity contamination. As shown in Fig. S1, the mid-visible fluorescence spectra of the sample are a strong function of excitation wavelength, suggesting that more than one compound is contributing to the fluorescence observed when the excitation wavelength is changed within the normal porphyrin Soret absorption region. The double maximum in the emission spectrum obtained when exciting at 460 nm (as reproduced in Fig. 2 of the article) is assigned by the authors to being either (i) “… *influenced by the experimental conditions being the multiple vibrational levels of the electronic transition more or less resolved.”* or (ii) *“… the result relative to absorption of the Soret band that is being provided by two main transitions.”* Neither of these suggestions are commensurate with the spectroscopy and photophysics of pure metalloporphyrins containing d^0^ or d^10^ metal ions of low atomic mass. A rigid macrocyclic structure such as that of the desired PETBP molecule in solution at room temperature will not exhibit two Soret vibrational bands of roughly equal intensity under any spectral resolution. Reduction of the macrocycle symmetry from *D*_4h_ does remove the near degeneracy of the Soret transition, but if the two transitions are to be observed in the emission spectrum they surely should also be seen in the absorption spectrum, but they are not. Unfortunately, the authors do not report fluorescence excitation spectra that could identify the absorption spectra attributable to each of their reported fluorescence bands.

Finally, one should note the differences in the spectra when the dye is anchored to TiO_2_ or ZrO_2_. If the labelling of the absorption spectra of Fig. 1 is correct, the absorption spectrum of the dye in the Q band region when anchored to the semiconductor does not change much, but is substantially narrower in the Soret region. The authors claim incorrectly that the absorption spectra broaden. The Q band emission spectra (Fig. 2) do broaden, as expected, when anchored to both TiO_2_ and ZrO_2_, but this is not true of the absorption spectra (if the labelling is correct). A narrowing of the dye's absorption spectrum, only in the Soret region, when anchored to a semiconductor surely must indicate that more than one compound is contributing to the absorption in the solution spectra in the Soret region. When the desired compound, PETBP with its carboxyl anchor, is selectively bound to the semiconductor, this results in a narrower spectrum despite being bound in the solid state because the contaminant is not anchored.

## Conclusions

The need for new, broadly absorbing, photostable dyes that can be incorporated into dye-sensitized solar cells is clear, and the compound synthesized by the authors for this purpose, PETBP, may be of some interest. However, the observed spectra, dynamics and the implied resulting photophysics reported by the authors cannot be attributed solely to the intended compound. The reported data can be particularly misleading if researchers are looking for metalloporphyrins that have rather long-lived upper excited electronic states and thus can act as dual absorber-upconverters.^6^

## Conflicts of interest

There are no conflicts to declare.

## Supplementary Material
